# The field of expertise modulates the time course of neural processes associated with inhibitory control in a sport decision-making task

**DOI:** 10.1038/s41598-022-11580-3

**Published:** 2022-05-10

**Authors:** Marie Simonet, Paolo Ruggeri, Etienne Sallard, Jérôme Barral

**Affiliations:** 1 Institute of Sport Sciences, University of Lausanne, Lausanne, Switzerland; 2 Brain Electrophysiology Attention Movement Laboratory, Institute of Psychology, University of Lausanne, Lausanne, Switzerland

**Keywords:** Cognitive neuroscience, Sensorimotor processing

## Abstract

Inhibitory control (IC), the ability to suppress inappropriate actions, can be improved by regularly facing complex and dynamic situations requiring flexible behaviors, such as in the context of intensive sport practice. However, researchers have not clearly determined whether and how this improvement in IC transfers to ecological and nonecological computer-based tasks. We explored the spatiotemporal dynamics of changes in the brain activity of three groups of athletes performing sport-nonspecific and sport-specific Go/NoGo tasks with video footages of table tennis situations to address this question. We compared table tennis players (n = 20), basketball players (n = 20) and endurance athletes (n = 17) to identify how years of practicing a sport in an unpredictable versus predictable environment shape the IC brain networks and increase the transfer effects to untrained tasks. Overall, the table tennis group responded faster than the two other groups in both Go/NoGo tasks. The electrical neuroimaging analyses performed in the sport-specific Go/NoGo task revealed that this faster response time was supported by an early engagement of brain structures related to decision-making processes in a time window where inhibition processes typically occur. Our collective findings have relevant applied perspectives, as they highlight the importance of designing more ecological domain-related tasks to effectively capture the complex decision-making processes acquired in real-life situations. Finally, the limited effects from sport practice to laboratory-based tasks found in this study question the utility of cognitive training intervention, whose effects would remain specific to the practice environment.

## Introduction

Inhibitory control (IC) refers to the ability to stop motor or cognitive processes^1^. While training IC has been shown to reduce the response time (RT) in the trained task and to induce functional changes within IC-related brain areas^2–4^, the question of whether and how the effects of IC training transfer to untrained computer-based tasks remains unanswered.

Current literature on computer-based cognitive training has suggested that regularly practicing difficult IC tasks may favor a transfer to untrained tasks^5–7^. Functional studies showed that IC shares common neural nodes with other cognitive processes^8–12^ and that modifications within this IC domain-general network would in turn influence the other supported functions (*for a review see* Spierer et al.^13^). Although an increasing task complexity may lead to the recruitment of combining several closely related executive functions, some authors reported no or small transfer effects following IC training^14^. For instance, Simonet et al.^14^ showed that 10 days of complex executive control training led to functional changes within IC domain-general prefrontal areas without transfer effects to other cognitive tasks. Similarly, a 3-week training with Go/NoGo and Stop-signal tasks did not result in larger transfer effects on untrained tasks compared to an active control group^15^. These papers suggest that the effects of IC training in a controlled laboratory environment would not necessarily transfer to other tasks. One might thus wonder whether IC training in a real-life environment would lead to transfer effects on nonecological and ecological IC tasks, supposing that the high complexity of this environment would functionally or structurally reshape IC domain-general areas.

Neurophysiologically, the long-term practice of IC assessed in specific populations facing complex situations has been documented to induce functional changes within frontal areas (e.g., elite fencers^16^; baseball players^17^), dorsomedial frontal and parietal areas (e.g., fighter pilots^18^) and within the cingulate cortex (e.g., elite fencers^19^). In terms of time, inhibitory processes have been shown to manifest approximately 200 to 500 ms after stimulus onset^2,20^. This time period, corresponding to the N2 and P3 event-related potential (ERP) components^21^, encompasses processes ranging from premotor inhibitory control^22^ to the cognitive implementation of the stopping action^2,23^. With regard to the differences between expert and control populations, Di Russo et al.^19^ used a Go/NoGo task and identified that fencers elicited larger N2 and P3 peak amplitudes than controls in NoGo trials. Similarly, higher N1, N2 and P3 peak amplitudes were observed for fencers than for nonathletes, with no difference with boxers^24^. While these studies have focused on the peak amplitude of the components, Wessel and Aron^25^ linked the P3 onset latency with successful inhibition and suggested that the timing of the components potentially represents a better indicator of the speed and success of response inhibition than focusing on their peaks.

Sport, given its dynamic nature, the ongoing interaction between different performers and the time constraints under which decisions must be made, represents an ideal vehicle to investigate the effects of long-term IC training in complex situations. Athletes practicing an open-skill sport (e.g., tennis, fencing, and soccer) requiring players to behave in a continuously changing environment^26^ have recorded faster RT in Go/NoGo tasks^16,24,27^, as well as changes in brain structure and function within the IC network^16,19,26,28–31^ compared to nonathletes. On the other hand, athletes practicing closed-skill sports (e.g., swimming, high jump, and ballet) requiring performance in a predictable and stable environment face only a few situations involving IC and exhibit lower performance on IC tasks compared to athletes practicing open-skill sports^26^.

Although athletes who have practiced an open-skill sport for several years appear to display better IC performance on standardized computer-based IC tasks designed with various types of stimuli than nonathletes (letters^16^; vertical and horizontal bars^19^; color squares^27^; various forms^29^), whether and how this expertise transfers to other more ecological tasks involving domain-specific stimuli or domain-specific strategies remains unclear. Only one study has recently shown that expert baseball players were faster than novices on a Go/NoGo task involving real scenarios of simulated baseball trajectory^17^. Interestingly, a vast body of evidence from studies employing more ecological tasks with video footage of sport situations exists, but all these studies have focused on perceptual-cognitive expertise^32–34^, leaving the assessment of IC in ecological tasks underinvestigated.

In this context, electrical neuroimaging analyses of ERP were chosen to identify the brain dynamics underlying the effects of years of practice in open- versus closed-skill sports on nonecological and more ecological Go/NoGo tasks. We included experts in table tennis and basketball since these sports represent an interesting model to investigate IC as they constantly require reacting to the opponent’s feints and suppressing programmed actions. A control group composed of closed-skill sports endurance athletes was added to control for the mere effects of practicing a sport. The three groups performed one sport-nonspecific Go/NoGo task with letters and one more ecological sport-specific Go/NoGo task with video footage of table tennis situations. We first assume that larger transfer effects on the sport-nonspecific Go/NoGo task would be achieved by experts in table tennis and in basketball than the endurance athletes. Second, we expect the table tennis experts to show better performance on the sport-specific Go/NoGo task than the two other groups because they would use their own motor expertise to facilitate the processing of postural information picked up from the opponent.

Electrophysiologically, since the latency of the ERP components has been identified as a reliable indicator of the speed of response suppression^25^, the topographic analyses of variance (TANOVA) and microstate analyses were used to explore the spatiotemporality of events. We hypothesized that the three groups would engage in difference perceptual-cognitive strategies. We assume that the expected faster RT of the table tennis group would be supported by an earlier recruitment of frontal and parietal cortices as these regions have been shown to support IC processes^14,35,36^ and to be involved in the action observation network^37,38^. This network would be modulated by the table tennis players’ expertise with the observed actions.

## Methods

### Participants

We included 20 table tennis players who played at a Swiss national club level, 21 basketball players who played at a Swiss national club level and 21 endurance players, who volunteered to participate (Table 1 for details). Participants reported normal or corrected-to-normal vision and no history of neurological or psychiatric disease. For the behavioral analyses, participants whose mean RT or mean percentage of error was < 2 or > 2 standard deviations from the group mean RT or percentage of error were excluded (*n* excluded: 5 participants; participants excluded based on the mean RT *n* = 2; participant 1 mean = 475.5 ms, median = 472.0 ms, participant 2 mean = 480.5 ms, median = 477.0 ms, group mean = 364.5 ms, group SD = 43.9). We included right- and left-handed participants, because all participants responded with their dominant hand. For the electroencephalography (EEG) analyses, we included only right-handed participants^39^, among whom we excluded three more participants due to bad EEG signals. We included only participants with a minimum of 70 trials comprising the ERP for the NoGo condition and 80 trials comprising the ERP for the Go condition in each task. We kept only right-handed participants in the EEG analyses, because left-handedness has been associated with regional brain asymmetry in comparison to right-handedness^40^. Written informed consent was obtained from all participants prior to the investigation. The experimental protocol and the methods were approved by our local ethics committee (Vaud, Switzerland; protocol N°2018/01803) and were conducted in accordance with the code of ethics of the World Medical Association (Declaration of Helsinki) for experiments involving human subjects in research.

**Table 1:** Characteristics and demographic data of the groups.

	Table tennis	Basketball	Endurance
Total participants	20	21	21
Women (w)	2	7	8
Left-handed	5	6	0
Age (years)	24.6 ± 3.7	24.6 ± 3.2	27.2 ± 4.7
Years of practice	13.7 ± 3.6	15.7 ± 4.4	12 ± 6.4
Hours per week	7.9 ± 2.9	10.8 ± 4.1	9.6 ± 6.7
Participants—behavior	20 (2 w)	20 (7 w)	17 (5 w)
Participants—EEG	14 (1 w)	14 (4 w)	16 (5 w)

Age, years of practice and hours per week are reported as the means ± standard deviations. Participants—behavior and participants—EEG refer to the number of participants included in the behavioral and EEG analyses, respectively.

### Procedure and tasks

The participants were seated in a quiet dark room facing a computer screen (Dell, 1707FPt 17’’ Flat Panel Monitor, Texas, USA) placed 50 cm from their eyes. They performed the sport-specific and sport-nonspecific Go/NoGo tasks one after another. The order was randomized across participants to avoid learning effects. The completion of each task lasted approximatively 25 min in total. Stimulus delivery was monitored using E-Prime 2.0 software (Psychology Software Tools, Inc., Sharpsburg, PA; http://www.pstnet.com/products/e-prime). A QWERTY keyboard was used as the response tool to record the RT. For the sport-specific task, the videos were recorded by a Canon camera (EOS 1200D model) at a frequency of 25 images per second and were processed with iMovie software (Apple, 2001–2018, Version 10.1.10).

#### Sport-nonspecific task

**Figure 1:**
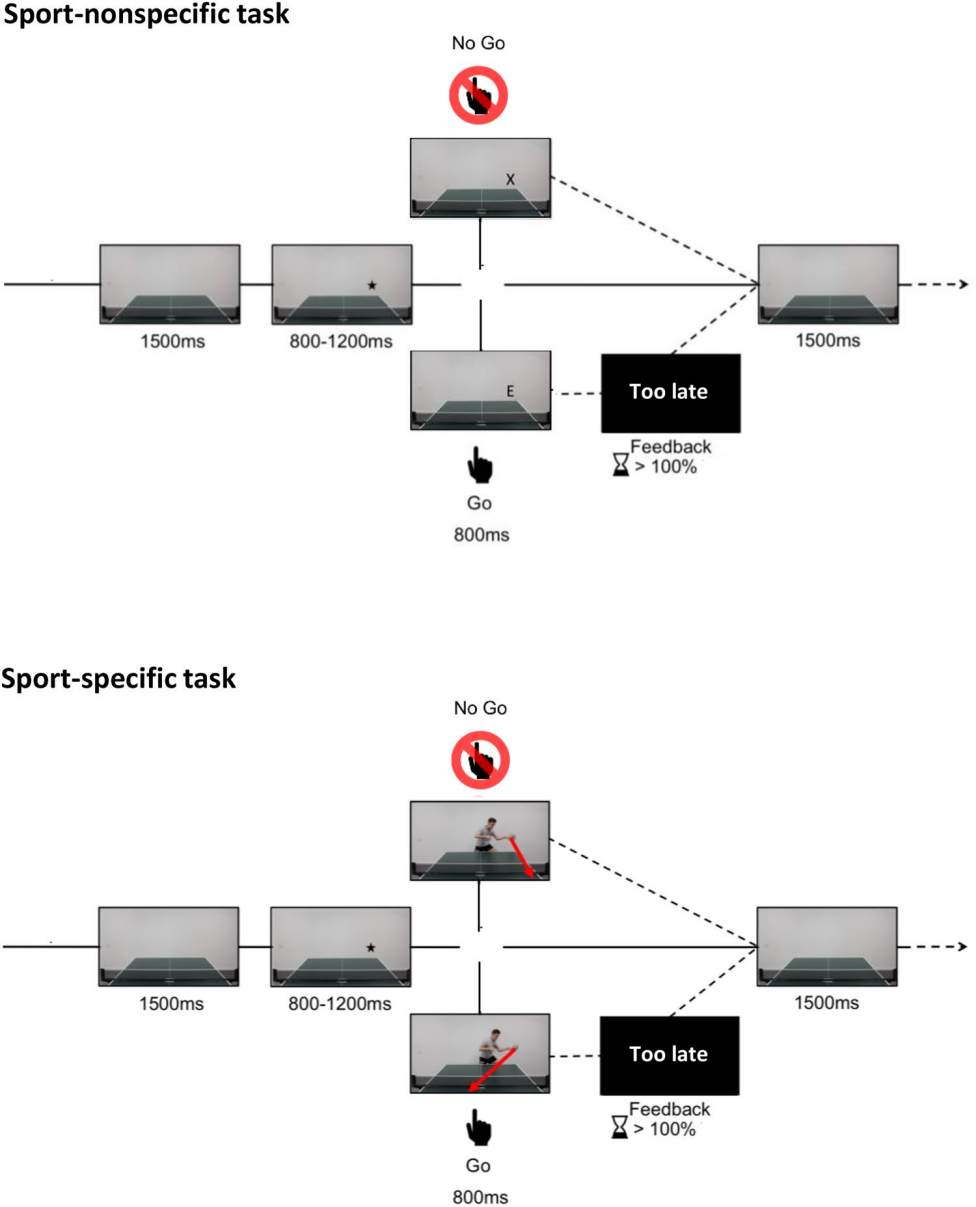
The experimental design of the sport-nonspecific and sport-specific Go/NoGo tasks. For the sport-specific Go/NoGo task, the stimuli are video footages of table tennis situations.

The sport-nonspecific task was a classical motor inhibition task. Participants first performed a training block consisting of 16 trials to familiarize them with the task’s instructions. A ‘calibration’ phase of 28 trials was completed to estimate the average RT of the participant to Go stimuli (for a similar procedure, see De Pretto et al.^41^; Simonet et al.^14^). A RT threshold corresponding to the average RT in the calibration phase was implemented in the following blocks so that feedback message of “too late” was displayed on the screen when the participant’s RT exceeded this RT threshold. A trial started with a table tennis table presented for a duration of 1500 ms (Fig. 1). Then, a black star was displayed either on the right or on the left corner of the table for a duration that randomly varied between 800 and 1200 ms. A letter chosen among A, E, I, L, N, O, R, T, U, X was then presented for 800 ms on the same side of the black star, and participants were instructed to press the spacebar on the keyboard as fast as possible with the right index finger in response to any letter except “X” (for the blocks where “X” was the NoGo stimulus) or “A” (for the blocks where “A” was the NoGo stimulus). The black star was implemented to diminish the artefacts due to eye movements in the EEG signal. The star was used to indicate the location of the upcoming stimulus to ensure that the participant stares at the correct location on the screen. The task included 4 blocks of 75 trials with a stimulus probability of 0.7 for the Go stimulus and 0.3 for the NoGo stimulus. The order of the presentation of the Go and NoGo stimuli was randomized. In the first two blocks, the black star was presented on the right side, and the NoGo stimulus was the letter “X” for the first block and “A” for the second block. In the third and fourth blocks, the black star was presented on the left side, and the NoGo stimulus was the letter “X” for the third block and “A” for the fourth block. Inhibitory control performance was assessed by recording the RT to Go stimuli and by measuring the false alarm (FA) rate (i.e., the percentage of errors).

#### Sport-specific task

The videos of the sport-specific task were created using a left-handed expert table tennis player (age: 20 years; years of practice: 13). A left-handed player was chosen to increase the difficulty of the task for the table tennis players, as they are less trained against this type of player. We assume that the basketball players and the endurance athletes would not notice the difference between a left- or a right-handed player, and therefore this left-handed opponent will only create more challenge for the table tennis players. A camera was positioned centrally behind the table tennis table so that it represented the typical viewing perspective when playing. Several sequences were recorded either when playing a forehand topspin or a backhand topspin. We selected several different sequences, namely, 31 forehand topspin sequences and 20 backhand topspin sequences, to avoid familiarization with repeated clips. All clips started at the moment when the ball was hitting the racket and were occluded 480 ms after the impact. The last image was frozen for 320 ms, and the clips lasted for 800 ms in total.

For the sport-specific task, participants first performed a training block consisting of 16 trials (8 trials forehand and 8 trials backhand) to familiarize them with the task’s instructions. A ‘calibration’ phase of 28 trials (14 trials forehand and 14 trials backhand) was completed to estimate the average RT of the participant to Go stimuli. An RT threshold corresponding to the average RT in the calibration phase was implemented in the following blocks so that feedback message of “too late” was displayed on the screen when the participant’s RT exceeded this RT threshold. A trial started with a table tennis table presented for a duration of 1500 ms (Fig. 1). Then, a black star was displayed either on the right corner or on the left corner of the table for a duration that randomly varied between 800 and 1200 ms. Then, the video clips started for 800 ms, during which participants were instructed to (i) press the spacebar of the keyboard as fast as possible with the right index finger when the ball was hit down the line and stop their response when the ball was hit cross court (rule 1 “cross court” was the NoGo stimulus) or (ii) press as fast as possible with the right index finger on the keyboard’s spacebar when the ball was hit cross court and stop their response when the ball was hit down the line (rule 2 “down the line” was the NoGo stimulus). The player always appeared on the same side of the star. The star was implemented to diminish the artefacts due to eye movements in the EEG signal. The star was used to indicate the location of the upcoming stimulus to ensure that the participant stares at the correct location on the screen. In position 1, the player appeared on the left of the table and therefore was playing forehand. In position 2, the player appeared on the left and therefore was playing backhand. To avoid learning effects, participants performed two blocks with position 1 rule 1, two blocks with position 2 rule 1, two blocks with position 1 rule 2 and two blocks with position 2 rule 2 alternatively. In total, the task included 8 blocks of 38 trials with a stimulus probability of 0.7 for the Go stimulus and 0.3 for the NoGo stimulus. The order of the presentation of the Go and NoGo stimuli was randomized. Inhibitory control performance was assessed by recording the RT to Go stimuli and by calculating the FA rate.

### Behavioral analyses

The required sample size of 60 participants was estimated with the G x Power^42^ software based on previous literature on skill-based differences with corresponding analyses^14,26,38^ to reach a power of 0.8 to detect a type I error of 0.05 and an effect size *f* 0.4 (i.e., large effect) with a one-way ANOVA of the three groups. Statistical analyses of behavioral data were performed using the jamovi project (2021)^43^. We used a free web application (http://www.estimationstats.com) to display the effect size of our results^44^. Prior to the analyses, the RT was subject to a procedure excluding trials < 100 ms and > 2 standard deviations from the individual’s mean RT. At the end of this procedure, the mean RT of each participant was computed with at least 90% of all Go trials for each task. First, RT and FA values were calculated for each participant. Then, the Shapiro–Wilk test was used to test for normality of the distribution of RT and FA values and Levene’s test was used to test for the equality of variances. For each task (sport-nonspecific and sport-specific), we computed a one-way ANOVA to compare the mean RT and FA of the three groups (table tennis, basketball, and endurance). A Tukey adjustment was employed to correct for multiple comparisons. Statistical significance was set to *p* < 0.05.

### EEG recording and data preprocessing

EEG data were acquired at a sampling rate of 1024 Hz with a 64-channel Biosemi Active two amplifier system (Biosemi, Amsterdam, Netherlands) and preprocessed using Brain Vision Analyzer 2 (Brain product, Munich, Germany, version 2.1). TANOVA and the microstate analyses were computed with the open-source software Randomization Graphical User interface (RAGU)^45^ based on MATLAB (http://www.mathworks.com/). Source analyses were performed with the Cartool software^46^ (Version 4.5.0).

After 512 Hz downsampling and filtering (0.31–40 Hz bandpass filter, DC removed), eye movement artifacts were corrected using independent component analysis. Electrodes displaying signal artifacts were interpolated using 3D splines^47^, leading to an average of 1.5% interpolated electrodes. For both sport-specific and sport-nonspecific tasks, the epochs were segmented from 100 ms prior to the stimulus to 800 ms after stimulus onset separately for Go and NoGo stimuli. A semiautomatic artifact rejection method was implemented to retain correct Go and NoGo epochs with an ERP amplitude within the ± 100 μV artifact rejection criterion. All epochs were then visually inspected after semiautomatic rejection, and the remaining artifact-containing epochs were removed. An average of 187 Go and 82 NoGo epochs for the sport-nonspecific task and 188 Go and 82 NoGo epochs for the sport-specific task were included in the statistical analyses. We averaged the epochs and applied a baseline correction (from − 100 ms to the stimulus onset), and the data were finally recomputed against the average reference. The averaged ERP waveforms can be found in the Supplementary Information (see Supplementary Fig. S1).

### ERP analyses

#### Test of topographic consistency

We computed the topographic consistency test (TCT) for each task (sport-specific and sport-nonspecific), stimulus (Go and NoGo) and groups (table tennis, basketball and endurance athletes) to determine the periods displaying a consistent pattern of source activity across subjects with the RAGU software, thus preventing the selection of time windows with inconsistent source activation across subjects^48^. The TCT was applied on the preprocessed ERPs computed from 0 to 800 ms after the stimulus onset and was run with 10,000 randomizations and a *p*-value threshold of 0.05. We performed the global duration statistics test and considered only continuous periods displaying a significant difference to control for multiple comparisons and to test whether the duration of the significant time period exceeded chance^48,49^ (for a detailed procedure, see Ruggeri et al.^50^).

#### Topographic analyses

TANOVA is a nonparametric randomization test that computes the global dissimilarity of the whole electrical field topographies between conditions and/or groups and assesses the significance of these topographic differences at each time point. We performed TANOVA for each task (sport-nonspecific task and sport-specific task) and each condition (Go and NoGo) separately to identify the periods showing significant topographic differences between the groups (table tennis, basketball, and endurance)^51,52^. The analyses were performed on the average ERP from 0 to 800 ms after stimulus onset. Using a nonparametric randomization test, this analysis assesses global dissimilarities in the whole electric field between the groups at each time point.

TANOVA was computed on amplitude-normalized maps (global field power (GFP) = 1) to obtain results that were independent of the GFP. The GFP is used to quantify the strength of a scalp potential field and is defined as the mean difference in potential between all possible pairs of electrodes^53,54^. We performed this normalization to identify the significant topographic differences between the groups (i.e., table tennis, basket, and endurance) that were independent of the global field strength. Ten thousand randomizations were conducted with a *p*-value threshold of 0.05. We performed the global duration statistics test to control for multiple comparisons^45,48,49^. This process tests whether the observed differences in a significant time period exceeded chance. Post hoc channelwise *t*-tests (*t*-maps) were conducted for statistically significant periods of interest (POIs) to further investigate the topographic distribution of the observed differences between groups.

#### Microstates

The microstate analysis and the source estimations were run when differences were found with the TANOVA analysis. The microstate analysis is based on the assumption that stable states may vary in their duration and strength of activation between conditions or groups (depending on the experimental design)^52,54,55^. This analysis decomposes the ERP signal to generate a set of stable, temporally ordered, topographic map configurations. The analyses were performed on the grand average ERPs of all subjects for each group from 0 to 800 ms after stimulus onset. A cross-validation procedure was applied 250 times by testing templates with 3 to 10 microstate maps to identify an optimal number of microstate maps fitting our ERPs. The topographic pattern of each microstate map was identified using the k-means algorithm with 50 random initializations^56^. The cross-validation procedure estimates of the variance explained as a function of the number of microstate templates. The explained variance typically increases as the number of microstate templates increases until it reaches a point where the addition of microstate templates does not consistently change the explained variance. This point was chosen to determine the optimal number of microstate map templates. Values for the onset, offset, duration and GFP of each microstate map were statistically compared between groups for Go and NoGo conditions separately. The onset and offset represent the beginning and the end times of a given microstate, respectively, while the duration corresponds to the difference between the offset and the onset values. The GFP represents the strength of the signal across the scalp for a given microstate. The statistical analysis of microstate parameters was separately performed using randomization statistics with a between-subject factor (table tennis, basketball, and endurance) for Go and NoGo conditions, 10,000 randomization runs, and a *p*-value threshold of 0.05.

#### Electrical source estimations

We used a local autoregressive average (LAURA) distributed linear inverse solution to identify the source estimations responsible for the different topographical dynamics observed between the groups^57,58^. The solution space was based on a realistic head model and included 3005 solution points selected from a 6 × 6 × 6 mm grid of voxels distributed within the gray matter of the average brain of the Montreal Neurological Institute (MNI, courtesy of R. Grave de Peralta Menendez and S. Gonzalez Andino, University Hospital of Geneva, Geneva, Switzerland). *T*-tests were computed to compare the source generators underlying microstate maps for each condition separately (i.e., Go and NoGo). We considered only clusters showing a *p*-value < 0.01 and composed of at least 15 contiguous nodes (*K*_E_) to control for multiple tests.

## Results

### Behavioral results

**Figure 2:**
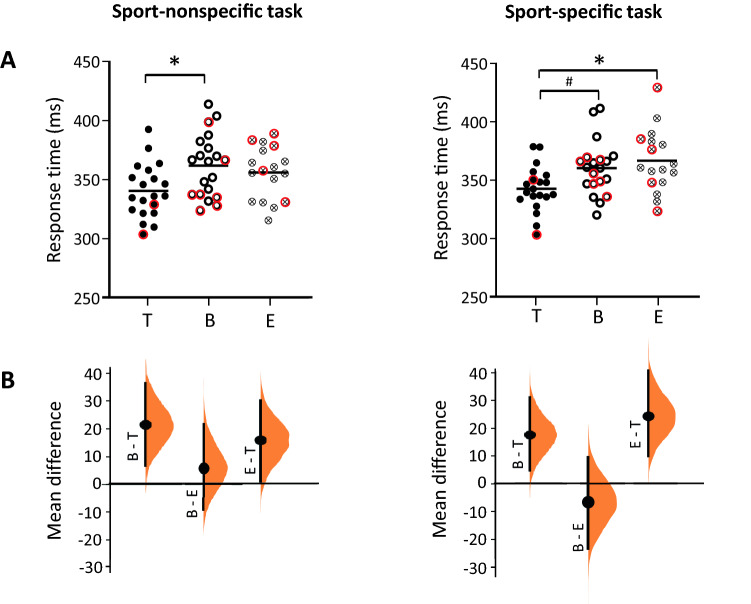
RT behavioral performance of the three groups on the sport-nonspecific and sport-specific Go/NoGo tasks. (**A**) The individual data (dots) and the mean values (horizontal line) are presented. The red circles indicate the performance of women. The asterisk (*p* < .05) and the hash (*p* = .05) indicate significant differences. (**B**) The mean difference between the groups is shown with the Gardner-Altman estimation plots^44^. The mean difference, representing the effect size, is plotted on a floating axis as a bootstrap sampling distribution. The mean difference is depicted as a dot; the 95% confidence interval is indicated by the ends of the vertical error bar. B = basketball; T = Table tennis; E = endurance. The graphs displayed in (B.) have been created on the website: http://www.estimationstats.com.

For the RT, a significant effect of Group on the sport-nonspecific task (F_2,54_ = 4.158; *p* = 0.024; η^2^ = 0.133) and the sport-specific task (F_2,54_ = 5.988; *p* = 0.006; η^2^ = 0.182) was observed (Table 2 and Fig. 2). In the sport-nonspecific task, the table tennis players responded significantly faster than the basketball players (t = 2.82; *p* = 0.018). In the sport-specific task, the table tennis players responded significantly faster than the endurance athletes (t = 3.16; *p* = 0.007) and the basketball players (t = 2.41; *p* = 0.050). The visualization of the effect size (Fig. 2B) added a qualitative explanation to the results presented in Fig. 2A. In the two tasks, the table tennis group showed an overall faster RT than the two other groups, as indicated by the higher mean difference between basketball-table tennis and endurance-table tennis groups compared to the mean difference between basketball-endurance groups.

**Table 2:** Behavioral data for the three groups’ performance and Welch’s ANOVA results for the sport-nonspecific task and the sport-specific task.

Mean ± SD	Sport-nonspecific task				Sport-specific task			
	Table tennis	Basketball	Endurance	Welch’s ANOVA	Table tennis	Basketball	Endurance	Welch’s ANOVA
RT (ms)	340.5 ± 22.8	362.0 ± 26.5	356.1 ± 22.3	*p* = .024 η^2^ = .133	342.7 ± 19.2	360.3 ± 23.3	366.8 ± 26.9	*p* = .006 η^2^ = .182
FA (%)	14.8 ± 6.6	13.6 ± 7.0	15.2 ± 6.9	*p* = .775 η^2^ = .009	21.1 ± 8.8	17.6 ± 10.0	20.1 ± 9.8	*p* = .504 η^2^ = .025

*RT* response time; *FA *false alarm. *P*-values and effect sizes are displayed for Welch’s ANOVA.

For the FA rate, we did not observe a significant effect of Group on the sport-nonspecific task (F_2,54_ = 0.257; *p* = 0.775; η^2^ = 0.009) or the sport-specific task (F_2,54_ = 0.699; *p* = 0.504; η^2^ = 0.025) (Table 2).

### ERP neuroimaging results

#### Topographic consistency test

The test showed significant and consistent topographies across the subjects of each group in the sport-specific and sport-nonspecific tasks within the time interval from 0 to 800 ms after stimulus onset. This result was validated by a significant global duration test. The TANOVA was thus performed between 0 and 800 ms.

#### Topographic analyses

##### Sport-nonspecific task

TANOVA revealed no significant topographic differences between the three groups.

##### Sport-specific task

**Figure 3:**
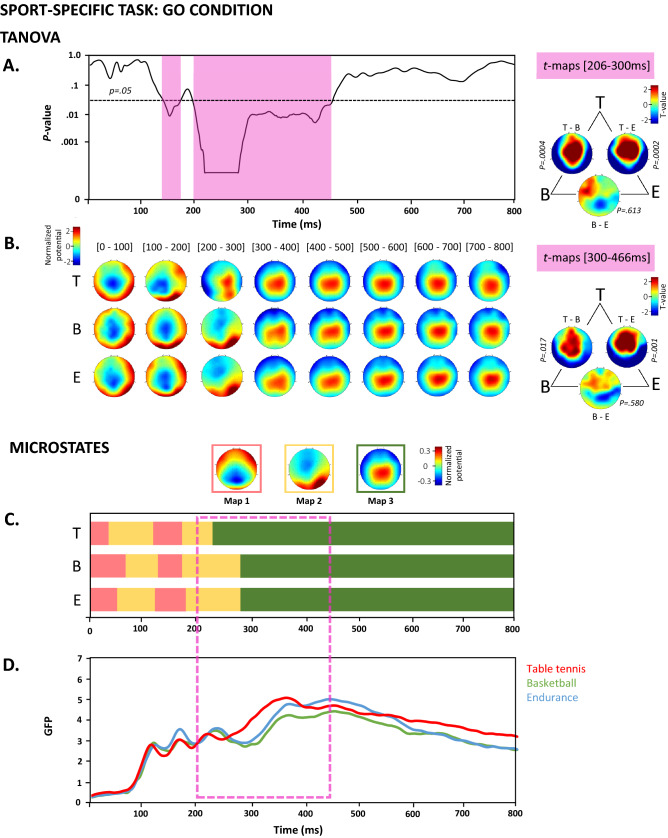
TANOVA, average topographies, *t*-maps and microstate analyses of the Go condition in the sport-specific Go/NoGo task. (**A**) The curve represents the *p*-values from the TANOVA plotted for each time point from 0 to 800 ms after stimulus onset. The period of significant topographic differences (*p* < .05) is highlighted in pink. (**B**) Mean ERP topographies were computed for each group separately over 100 ms time intervals. The topographies were normalized (GFP = 1). Red and blue indicate positive and negative potential values, respectively. The *t*-map contrasting each group with the other groups is shown for the POI divided into two time periods, namely, from 206 to 300 ms and from 300 to 466 ms. Positive (in red) and negative (in blue) t-values indicate more positive and more negative potentials regarding the order of the group contrast (T-E indicates the data from the table tennis group were compared to the data from the endurance group). The *p*-values from the TANOVA of the *t*-maps are displayed. (**C**) The topographies of the three microstate maps obtained from the cross-validation procedure and their duration are displayed for the three groups. Red and blue indicate positive and negative potential values, respectively. The topographies were normalized (GFP = 1). The colored frame surrounding each map corresponds to their occurrence in the graph below. (**D**) The GFP of the microstate maps is displayed as a function of time for each group. B = basketball; T = Table tennis; E = endurance. The topographies displayed in (**A**), (**B**) and (**C**) have been generated via MATLAB (http://www.mathworks.com/).

**Figure 4:**
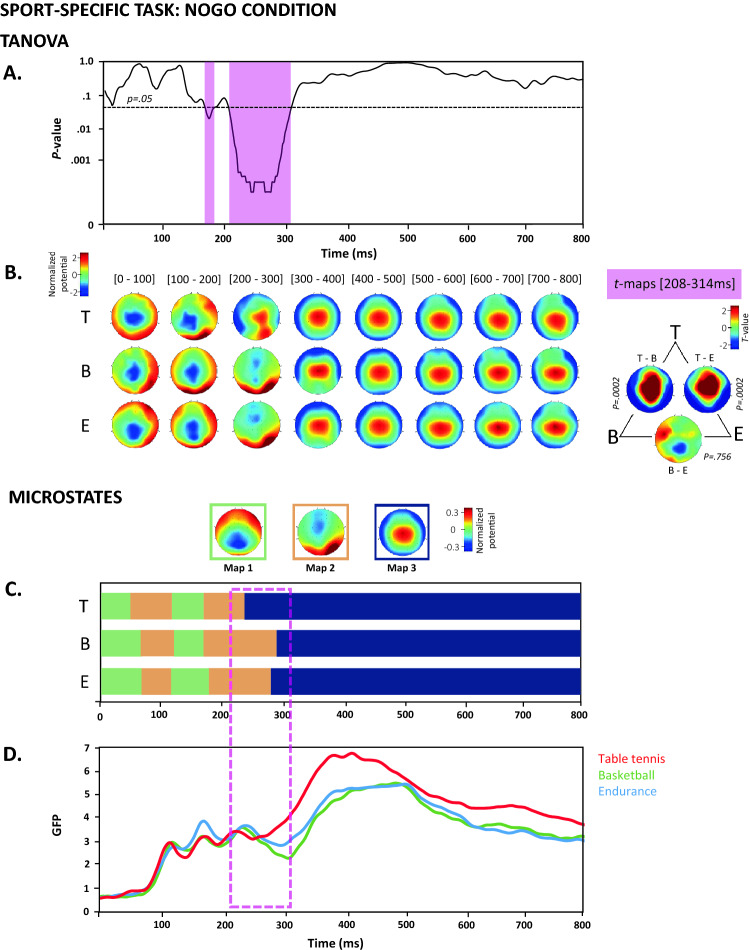
TANOVA, average topographies, *t*-maps and microstate analyses of the NoGo condition in the sport-specific Go/NoGo task. (**A**) The curve represents the *p*-values from the TANOVA plotted for each time point from 0 to 800 ms after stimulus onset. The period of significant topographic differences (*p* < .05) is highlighted in pink. (**B**) Mean ERP topographies were computed for each group separately over 100 ms time intervals. The topographies were normalized (GFP = 1). Red and blue indicate positive and negative potential values, respectively. The *t*-map contrasting each group with the other groups is shown for the POI. Positive (in red) and negative (in blue) t-values indicate more positive and more negative potentials regarding the order of the group contrast (T-E indicates that the data from the table tennis group were compared to the data from the endurance group). The *p*-values from the TANOVA of the *t*-maps are displayed. (**C**) The topographies of the three microstate maps obtained from the cross-validation procedure and their duration are displayed for the three groups. Red and blue indicate positive and negative potential values, respectively. The topographies were normalized (GFP = 1). The colored frame surrounding each map corresponds to their occurrence in the graph below. (**D**) The GFP of the microstate maps is displayed as a function of time for each group. B = basketball; T = Table tennis; E = endurance. The topographies displayed in (**A**), (**B**) and (**C**) have been generated via MATLAB (http://www.mathworks.com/).

TANOVA revealed significant topographic differences (*p* < 0.05) between groups from 206 to 466 ms for the Go condition (Fig. 3A) and from 208 to 314 ms for the NoGo condition (Fig. 4A). *T*-map contrasts were computed between the topographies of the three groups to quantify these differences (Fig. 3B and 4B). Under the Go condition, the time period with a significant difference lasted for more than 250 ms. We contrasted topographies in two consecutive time periods, between 206 and 300 ms and between 300 and 466 ms, to obtain more accurate insights into the temporal dynamics of the underlying processes. Between 206 and 300 ms, these contrasts revealed that the table tennis players were characterized by a more positive potential over central electrodes than basketball players and endurance athletes. The basketball players and endurance athletes did not exhibit specific differences in the topographies over this period. Between 300 and 466 ms, *t*-map contrasts revealed that the table tennis players were characterized by a more positive potential over frontocentral electrodes than basketball players and endurance athletes. The basketball players and the endurance athletes did not display specific differences in the topographies over this period.

Under the NoGo condition between 208 and 314 ms, these contrasts revealed that the table tennis players were characterized by a more positive potential over central electrodes than basketball players and endurance athletes. The basketball players and endurance athletes did not exhibit specific differences in the topographies over this period. The more positive potential over central electrodes presented by the table tennis in the different periods of interest could inversely be interpreted as a more negative potential of the basketball players and endurance athletes.

The statistical details of all the *t*-maps can be found in the Supplementary Information.

#### Microstates

The microstate analysis was computed for the sport-specific task, for which the TANOVA revealed periods of significant topographic differences between Go and NoGo conditions. For both the Go and NoGo conditions, the microstate analysis identified three maps that explained 80% of the ERP variance. The microstate maps and the GFP of the microstates are displayed in Fig. 3C and 3D for the Go condition and in Fig. 4C and 4D for the NoGo condition. In the Go condition, the statistics computed on microstate parameters revealed a significant effect of Group on microstate map 2 for the offset parameter (table tennis = 240 ms, basketball = 295 ms, endurance = 295 ms, *p* = 0.0001) and on microstate map 3 for the onset parameter (table tennis = 242 ms, basketball = 297 ms, endurance = 297 ms, *p* = 0.0001). In the NoGo condition, the statistics computed on microstate parameters revealed a significant effect of Group on microstate map 2 for the offset parameter (table tennis = 248 ms, basketball = 302 ms, endurance = 298 ms, *p* = 0.0002) and on microstate map 3 for the onset parameter (table tennis = 250 ms, basketball = 304 ms, endurance = 300 ms, *p* = 0.0009) and the mean GFP (table tennis = 4.26 µV, basketball = 3.09 µV, endurance = 3.30 µV, *p* = 0.0045). Interestingly, the period of significant differences revealed by TANOVA was compatible with the different latencies of the microstate maps. The underlying brain generators responsible for the shift in microstate map latencies in the table tennis group were further investigated using source estimations.

#### Electrical source estimations

**Figure 5:**
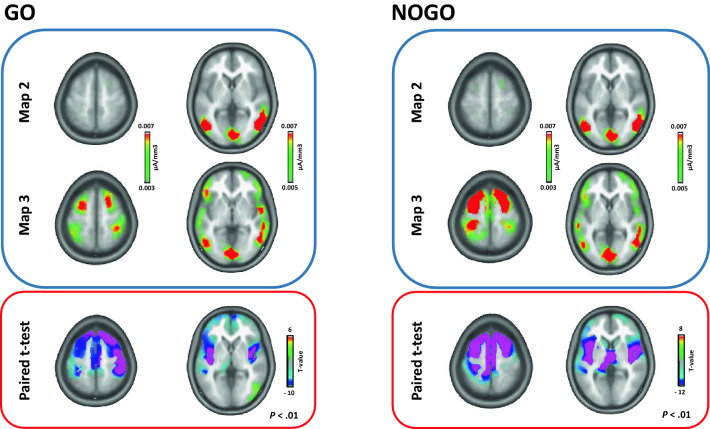
Source estimations for the GO and NOGO conditions comparing microstate map 2 and microstate map 3 in the sport-specific Go/NoGo task. The blue panel outline represents the mean activity for each condition (Go and NoGo) and each microstate map (map 2 and map 3). The red panel outline illustrates the regions showing between-map significance differences. Negative values (blue, violet) indicate the areas that presented greater involvement in microstate map 3. The brain slices have been generated with the Cartool software46 (Version 4.5.0).

Since we were interested in the spatiotemporal architecture of the IC processes, we compared the electrical source estimations for the microstate maps that showed significant differences in the onset or the offset parameters, namely, microstate map 2 and map 3 for the Go and NoGo conditions, respectively (Fig. 5). First, individual ERP data were averaged over a time period around the peak GFP of each group (± 25 ms from the peak) for microstate maps 2 and 3 to generate a single data point per microstate map for each participant and condition. Then, the sources were estimated for each participant and each microstate map. For the Go condition, the paired *t*-test revealed higher activity (*p* < 0.01, *K*_E_ > 15) for map 3 than map 2 within regions including the superior temporal gyrus (bilateral), precentral and postcentral gyri (bilateral), insula, inferior parietal lobule (bilateral), cingulate gyrus, superior and medial frontal gyri (bilateral), and right inferior frontal gyrus. For the NoGo condition, the paired *t*-test revealed higher activity (*p* < 0.01, *K*_E_ > 15) for map 3 than map 2 within regions including the superior and middle temporal gyri (bilateral), the precuneus, the cingulate gyrus, the inferior parietal lobule (bilateral), the precentral and postcentral gyri (bilateral), the middle and superior frontal gyri (bilateral), and the inferior frontal gyrus (bilateral).

## Discussion

We investigated the transfer effects of years of intensive practice in table tennis, basketball or endurance sports on computer-based nonecological and ecological Go/NoGo tasks. We assessed the behavioral performance (RT and FA) and the brain spatiotemporal dynamics indexing IC. Overall, the experts in table tennis responded faster than the two other groups with no difference in FA, which was supported by the earlier recruitment of brain regions involved in the execution or suppression of the motor response.

### Generalization effects: no clear pattern of transfer effects

At the behavioral level, the first assumption was that the table tennis and the basketball players would show better performance on the sport-nonspecific Go/NoGo task than the endurance athletes because practicing an open-skill sport would lead to transfer effects on a standardized IC task^4,26^. The table tennis players responded significantly faster than the basketball players, but no significant differences in either RT or FA were observed between the table tennis players and the endurance athletes or between the basketball players and the endurance athletes. To propose a more qualitative explanation, we showed the mean difference in RT between groups displayed a similar pattern of results between the basketball and table tennis players, as well as between endurance athletes and table tennis players (Fig. 2B). This result indicates that table tennis players would be globally faster than the two other groups in the sport-nonspecific Go/NoGo task, despite the lack of a significant difference obtained with traditional null hypothesis significance testing, which can restrict the interpretations that a researcher may draw as a result of adherence to the dichotomous outcome^44^. In contrast to other studies comparing the performance of expert athletes to nonathletes on IC tasks and showing that experts outperformed nonathletes^16,17,19,27,59^, a control group composed of endurance athletes was chosen. Aerobic or strength training has been shown to improve IC^60^, although to a lesser extent than practicing an open-skill sport^26^; therefore, assigning endurance athletes to the control group aimed to minimize the effect of sport practice. Based on our findings, we may have underestimated the positive effects of aerobic training or chronic physical activity on IC^61,62^. The results would rather suggest that the long-term practice of an endurance sport such as running, cycling, swimming or cross-country skiing led to the same level of IC proficiency as the practice of an open-skill sport when IC was assessed with a sport-nonspecific task. Finally, because the RTs of the three groups were relatively low when compared to other studies with a similar task design assessing nonathletes^4,14,16^, the practice of table tennis, basketball or endurance sports could have improved IC performance effectively and have led to transfer effects on a nonecological Go/NoGo task. The inclusion of a nonathlete control group would have helped disentangle the issue of whether the mere practice of a sport, either open- or closed-skill, improves IC on nonecological IC tasks.

In the sport-specific Go/NoGo task, the table tennis players responded significantly faster, while between-group differences in the FA rate were not observed. A faster RT with no change in FA has been previously shown to be an index of better IC performance. Race models indeed postulated that a higher speed of the execution process along with the same rate of errors is an indicator of improved inhibition processes^63,64^. We suggest that because experts in table tennis underwent years of intensive training in conflict situations in which fast suppression of planned and ongoing actions is required, they would arguably transfer their superior IC proficiency to other tasks related to their practice. Regarding the results of the basketball players, they did not present better IC scores than the endurance athletes for the sport-specific IC task, despite their expertise in dealing with complex and dynamic situations. In other words, the effects of the long-term practice of basketball did not generalize to a table-tennis-based task, which could be linked to the conclusions of previous studies showing small transfer effects following cognitive training when the tested tasks are not closely related to the trained tasks^65,66^. Also, because every sport has its own specific rules and requirements, such as the time constraints under which decisions must be made, the complexity of the surrounding environment, the number of teammates and opponents, the speed of the game, it would be too simplistic to consider open- or closed-skill sports as homogeneous groups. Even though table tennis and basketball belong to the same category of open-skill sports and both require the ability to deal with complex situations in dynamic environments, these two sports have their own specificity. This could explain the difference of performance between the basketball players and the table tennis players.

For this study, a sport-specific task with video footage of table tennis situations was designed, as we think that this task is best suited to capture the inhibition processes trained by table tennis players. Actually, a gap exists in the literature, as most studies used nonecological tasks to evaluate IC in expert populations^18,19,26,67^. One exception is the study by Muraskin et al.^17^, who compared baseball players to baseball novices with a Go/NoGo task reflecting real scenarios of simulated baseball trajectories. Consistent with our results, the authors showed that experts in baseball responded faster than novices^17^. The facilitation of the RT in baseball players compared to novices was also shown in previous studies using sport-nonspecific Go/NoGo tasks^68,69^. Although the Go/NoGo task developed by Muraskin et al.^17^ was designed to be in-game baseball-specific and is thereby more ecological than a standardized Go/NoGo task with letters, the stimuli (i.e., fixed images) remain too far from the real complex practice environment to identify sport-specific IC performance or sport-specific IC neural signatures. In the sport-specific task designed in this study, one might wonder whether perceptual-cognitive skills could play an important role in addition to inhibition processes. Since we built a dynamic and complex task with sport-specific situations, we can not exclude that the better performance of the table tennis players would also rely on their higher perceptual-cognitive skills related to table tennis situations^70^. It might be that they would be able to anticipate the outcome of the situation faster than the two other groups. In addition to higher IC performance, the better performance of the table tennis players in this sport-specific Go/NoGo tasks could also be explained by their higher expertise in anticipating table tennis situations.

### IC expertise is associated with a temporal shift in topographies when IC processes are implemented

In the table tennis sport-specific Go/NoGo task, between-group differences were observed in NoGo trials between 200 and 300 ms, a period corresponding to the N2 component^71^ previously associated with the detection and resolution of response conflict and the initiation of the IC processes^41,72–75^. In the Go trials, between-group differences manifested between 200 and 450 ms, a time period characterized by the occurrence of N2 and early P3 components^2^. The observed difference in the P3-related time period, i.e., between-group difference up to 450 ms, suggests that our groups differ significantly in late decisional and response initiation processes when a motor response is required. By combining the TANOVA and microstate analysis results, we observed an earlier transition from microstate map 2 to microstate map 3 in the table tennis group for the Go and NoGo conditions. Interestingly, P3-related topography displaying frontocentral positivity, as displayed in microstate map 3, has been previously associated with motor inhibition processes^73^ and might thus explain the better behavioral inhibition performance of the table tennis group.

The spatiotemporal shifts in brain mechanisms supporting IC performance have already been documented in other groups, such as in old compared to young adults^76^ or in patients with Parkinson’s disease compared to controls^77^, or when the complexity of the task was increased^74^, but this study is the first to report these spatiotemporal shifts in different groups of athletes. Few studies comparing athletes and nonathletes have investigated the latency and amplitude of the N2-related and P3-related NoGo components, but the authors restricted their analyses to a prior selection of specific electrodes and did not consider the whole topographic pattern of activity^19,24,29,59,69^. Bianco et al.^24^, for instance, presented evidence of earlier latency of the P3 component recorded at Fp1, Cz, PO7 sites in fencers compared to boxers, despite the similarities regarding the high attentional demand, the agility and the quick decision-making requirements of these two sports. Additional evidence comes from You et al.^29^ who compared the performance of table tennis players and nonathletes on conscious and unconscious Go/NoGo tasks. The authors showed decreased RT along with shorter N2 component latencies and larger P3 component amplitude in table tennis players than in nonathletes by analyzing frontocentral electrodes (i.e., Fz, FCz and Cz). In line with these results, substracted NoGo N2 peak latency (i.e. subtraction the averaged waveforms of Go trials from NoGo trials) measured at frontal and midline electrodes during a Go/NoGo task was demonstrated to be shorter in baseball players compared to track and field athletes^78^. Based on these previous studies, computing topographic analyses such as those presented in our study, allows us to define the onset and offset of the components and thus their latency, which might provide a better indication of the speed and success of response inhibition^25^.

With regard to the results of the sport-nonspecific Go/NoGo task, the topographic ERP analyses revealed no period showing significant differences between the three groups in the NoGo or Go condition. This result is consistent with the behavioral pattern of results that revealed no strong differences between the three groups. Compared to the sport-specific Go/NoGo task, we assume that the effect size may be smaller in this task, and thus the number of participants might be too small to reach the level of significance.

### IC expertise mainly relies on the earlier recruitment of regions involved in inhibition and decision-making processes

Contrasting the sources of microstate maps 2 and 3 provides insights into the brain regions responsible for the enhanced behavioral performance of the table tennis group. The analyses were performed for the Go and NoGo conditions separately, and interestingly, we found similarities in the location of the effects. Overall, the results revealed a greater involvement of frontotemporal areas, the cingulate gyrus, the inferior parietal lobule and the postcentral gyrus in map 3 than in map 2. Additionally, specific to the NoGo condition, the precuneus, a region previously associated with visuospatial attention^79^ and oculomotor inhibition^80^, was more activated in map 3 than in map 2. Specific to the Go condition, the insula, a region related to sensory-motor integration and especially to stimulus–response coupling to guide response selection^81–83^, exhibited greater activation in map 3 than in map 2. Overall, the localization of our effects is consistent with previous studies linking these regions with inhibition processes and also more generally with decision-making processes. In particular, while the superior temporal gyrus is a site of multisensory integration, especially of auditory and visual cues^84,85^, frontal areas and the cingulate gyrus have been shown to be involved in conflict processing and IC mechanisms^2,14,86–88^. Furthermore, the inferior parietal lobule has been shown to play a role in stimulus-driven attention and maintaining attentive control on task goals, as well as in action observation networks^89,90^. This result is consistent with previous reports showing a greater activation of this action observation network if the subject has prior experience with the action displayed^91^. As an example, Balser et al.^91^ documented greater activation of the action observation network in experts in tennis and volleyball when they were facing situations related to their sport compared to situations unrelated to their sport. Accordingly, the motor repertoire of our table tennis group would explain the fast recruitment of areas involved in this action observation network. In summary, and in parallel with the temporality of the microstate analyses, we claim that the earlier activation of regions including frontotemporal areas, the cingulate gyrus, inferior parietal lobule, precuneus, insula and postcentral gyrus in the table tennis group would support the better performance of the table tennis players on this video-based task than athletes in the other two groups.

Brain sources supporting these two conditions were contrasted to specifically identify the regions supporting the Go and NoGo conditions^92^. In the present study, the sport-specific Go/NoGo task was evidently more resource-demanding in terms of perceptual-cognitive skills and visuospatial attention. The neural activity engaged to support this attentional and perceptual-cognitive load might have masked the very specific neural mechanisms underlying IC. In an attempt to diminish the influence of the multiple visuo-attentional processes involved in this task and to focus only on IC processes, we contrasted the Go and NoGo conditions within the same time period (see Supplementary Fig. S2). Interestingly, the Go-NoGo contrast computed over the period corresponding to the late phase of decisional processes (i.e., microstate map 3) revealed higher activity within regions including the superior and medial frontal gyrus, the cingulate gyrus and the paracentral lobule under the NoGo condition than under the Go condition. The localization of this contrast is consistent with the previous literature showing that the medial and superior frontal areas and the cingulate gyrus support conflict processing and the successful suppression of inappropriate actions^14,86–88^. This result highlights that although these regions have been shown to be activated in the Go condition and would support some processes related to decision-making, they would be involved in the NoGo condition to a greater extent and would thus be more directly related to IC mechanisms.

### Implications

Due to the growing interest in improving cognitive performance in the sporting domain^93^, an understanding of whether IC is transferrable between tasks or sport situations has theoretical implications for cognitive psychology and is crucial in applied sport psychology. Although some research projects seek to implement more ecological environments for applied perspectives, the methods employed often confine the researcher in the laboratory. We encourage researchers to develop research protocols considering the complexity of the sporting environment. An interesting line of research would be to study the impact of perceptual-cognitive expertise or contextual expertise^94^ on inhibitory control performance in athletes. As a future experimental design, it would be interesting to present some sort of contextual information or longer kinematic information to investigate whether table tennis players would show higher inhibitory control proficiency than novices. By showing a video of 200 or 300 ms before the ball hit the racket or by presenting some information related to the context of the situation, we could demonstrate whether the expertise of the table tennis players to integrate the visual cues or the contextual information led the players to inhibit an inappropriate action faster than novices. In this sense, because the complexity of open-skill sports seems to recruit different executive functions^95^—not only inhibition—and because it has recently been demonstrated that motor inhibitory control processes would be supported by more general mechanisms of action updating^96^, there is a need to disentangle the complex interactions between executive functions such as updating, switching and inhibition, which are crucial for the practice of open-skill sports.

### Limitations

In the present study, the absence of movement-based responses and real opponents might be viewed as a limitation. The emergence of new technologies, such as mobile EEG^97^ or virtual reality^98^, would help us design experiments in sport-specific environments to take into account the complexity of these environments and to ensure greater fidelity in participant responses. The second limitation is the selection of the participants. Although we recruited only highly skilled athletes, we cannot exclude interindividual differences in sport expertise (years of practice, level, age at the beginning of practice, etc.). The data regarding the sport expertise reported in Table 1 were given by the participants and need to be interpreted as an estimation of the expertise. Furthermore, the question has been raised whether hereditary factors leading to a natural predisposition toward a certain sport might be responsible for interindividual differences. Exceptionally gifted individuals might manifest higher learnability, outstanding attitude or willingness to undergo hard training regimens^99^, and the differences reported in this paper might at least partially reflect some genetic differences. Finally, the perceptual familiarity of the table tennis players with the stimuli (i.e. a tennis tabletop) could represent a potential confounding factor in the results, as it might give an advantage, albeit small, for the table tennis players.

## Conclusions

The novelty of this experiment was to test expert athletes on a Go/NoGo task with real sport situations. Together with the previous literature, our collective results show that the long-term practice of a sport involving an IC component led to higher performance in computer-based Go/NoGo tasks. Our main behavioral finding corroborates previous evidence showing limited transfer effects following IC training protocols and thus supports the assumption advanced by the literature that cognitive training would remain specific to the practice environment in which they are acquired. Neurophysiologically, we have shown for the first time that IC expertise in athletes relies on the rapid access of a large functional network, including subtle modulations of neural nodes that support the decisional processes in a dynamic sport-specific Go/NoGo task. We encourage researchers conducting future studies to consider the complexity of sport-related situations in their experimental designs to understand why ‘brain training’ interventions aimed at improving cognitive skills have failed to yield consistent conclusions to date^65^.

## Supplementary Information

Supplementary Information.

## Data Availability

The datasets generated and analysed during the current study are available in Zenodo repository (https://zenodo.org/record/4946356#.YMdv4B8za70 and https://zenodo.org/record/6500222#.YmpLx-dBw2w).
