# Supplementary material for: The field of expertise modulates the time course of neural processes associated with inhibitory control in a sport decision-making task
Source: Sci Rep. 2022 May 10;12:7657. doi: 10.1038/s41598-022-11580-3 (PMC9090811; doi:10.1038/s41598-022-11580-3)

Supplementary Figure S1: ERP waveforms averaged for each group, each task and each condition.

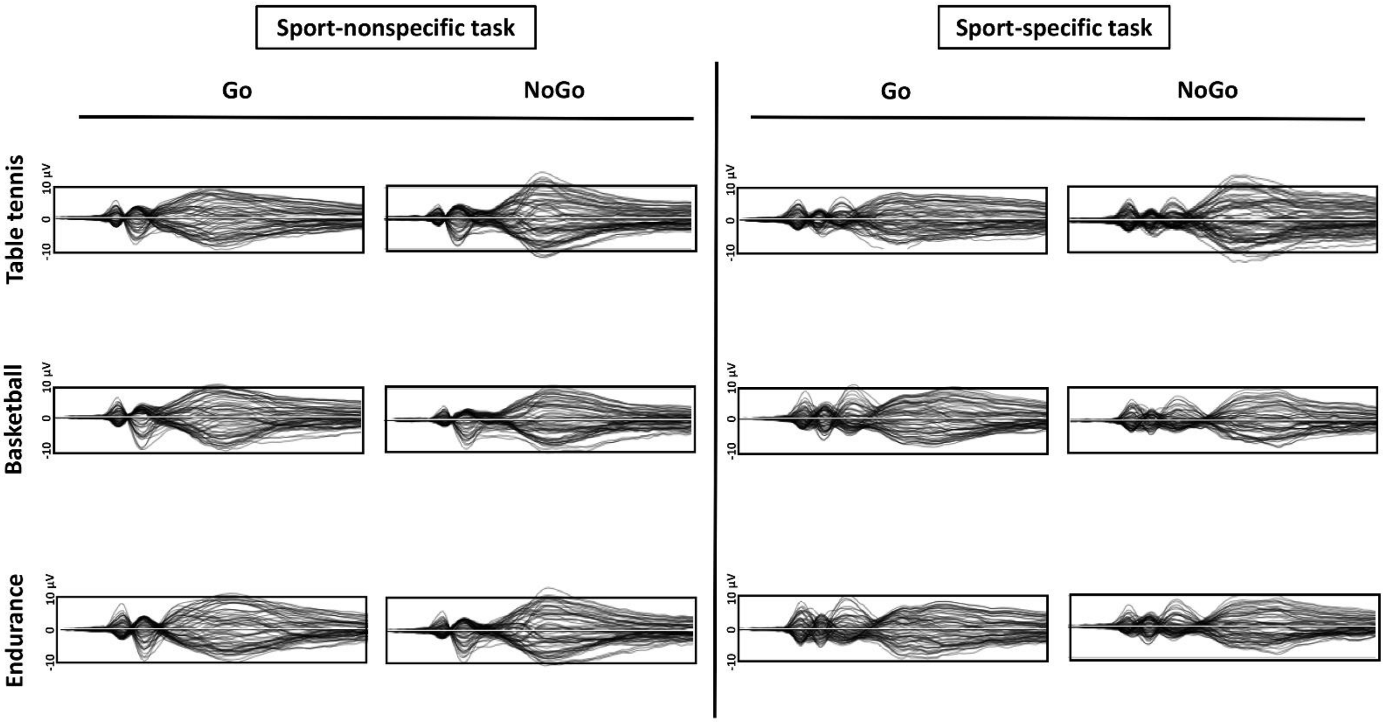

The averaged ERPs have been generated with the Cartool software[45] (Version 4.5.0).

Supplementary information on the t-maps of the sport-specific task.

In the Go condition, between 206 and 300 ms, these contrasts revealed that the table tennis players were characterized by a more positive potential over central electrodes than basketball players ($t_{max} = 4.64$ at electrode C2; $t_{min} = -4.87$ at electrode P9; $p = .0004$) and endurance athletes ($t_{max} = 5.30$ at electrode FC2; $t_{min} = -5.86$ at electrode P10; $p = .0002$). The basketball players and endurance athletes did not exhibit specific topographies over this period ($t_{max} = 2.448$ at electrode FC5; $t_{min} = -1.79$ at electrode Pz; $p = .6126$). Between 300 and 466 ms, *t*-map contrasts revealed that the table tennis players were characterized by a more positive potential over frontocentral electrodes than basketball players ($t_{max} = 2.83$ at electrode CP2; $t_{min} = -3.12$ at electrode P9; $p = .0174$) and endurance athletes ($t_{max} = 4.23$ at electrode F1; $t_{min} = -3.69$ at electrode P9; $p = .0014$). The basketball players and the endurance athletes did not display specific topographies over this period ($t_{max} = 1.5$ at electrode F3; $t_{min} = -2.11$ at electrode P2; $p = .5806$).

Under the NoGo condition between 208 and 314 ms, these contrasts revealed that the table tennis players were characterized by a more positive potential over central electrodes than basketball players (t_{max} = 4.23 at electrode CPz; t_{min} = −4.87 at electrode P9; p = .0002) and endurance athletes (t_{max} = 4.63 at electrode FC2; t_{min} = −4.89 at electrode P10; p = .0002). The basketball players and endurance athletes did not exhibit specific topographies over this period (t_{max} = 2.38 at electrode FC5; t_{min} = −1.80 at electrode Pz; p = .7458).

Supplementary Figure S2: The source localisation of the Go-Nogo contrast. Source estimations for the contrast between the GO and NOGO conditions within the time period corresponding to the microstate Map3 in the sport-specific Go/NoGo task. The blue shape presents the mean activity for each condition (G Map3 and NoGo Map3). The red shape illustrates the regions showing between-map significance differences. Negative values (blue, violet) indicate the areas that presented greater involvement in the NoGo condition. The brain slices have been generated with the Cartool software[45] (Version 4.5.0).

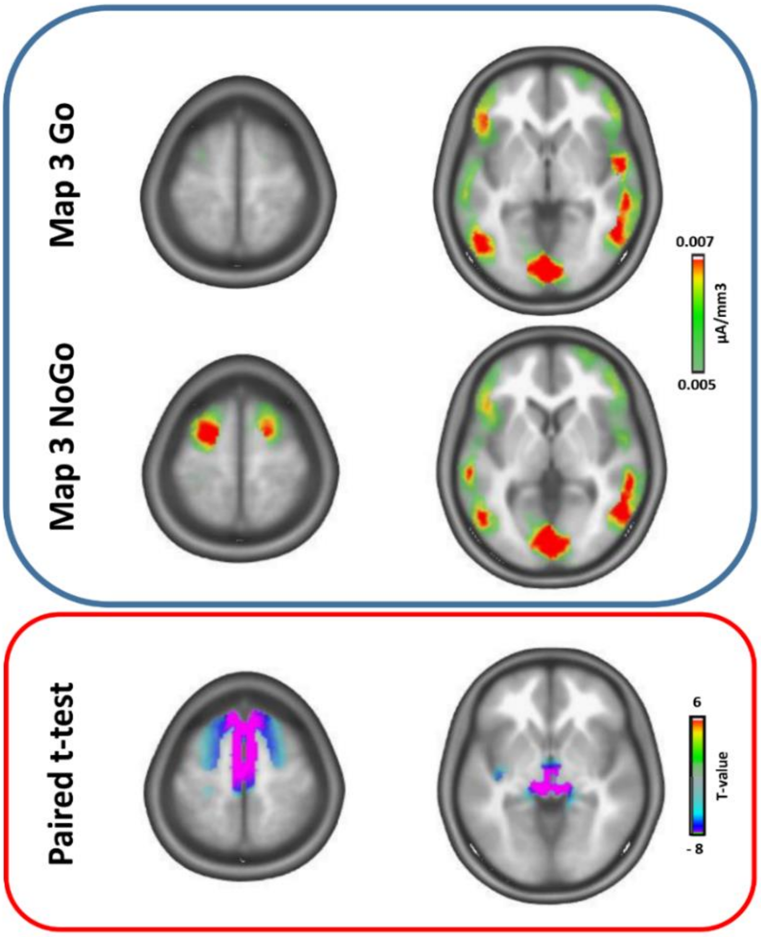

Supplement: Supplementary file 1 — Supplementary Information. [file 41598_2022_11580_MOESM1_ESM.pdf]
